# Letter in reply: Crusted scabies mimicking a lupus flare after rituximab and belimumab

**DOI:** 10.1016/j.jdcr.2021.07.041

**Published:** 2021-08-14

**Authors:** Grigoriy Androsov, Jonas A. Adalsteinsson, Diane Whitaker-Worth

**Affiliations:** Department of Dermatology, UConn Health, Farmington, Connecticut

*To the Editor:* We found the case report from August 2020 published in the journal by Ashrafzadeh and Layegh,^1^ concerning the association of rituximab with crusted scabies, to be informative. We want to reinforce this link through a patient encounter where a scabies infestation mimicked a lupus flare in a patient after rituximab and belimumab exposure, resulting in diagnostic delay.

A 28-year-old woman, with a history of systemic lupus erythematosus (SLE) with concurrent Sjogren syndrome, presented to the rheumatology department for evaluation of a worsening rash. Elevated serologies at time of diagnosis of SLE included antinuclear antibody titer (speckled pattern) at 1:2560, anti-Ro (SS-A) antibodies at 114, anti-La (SS-B) antibodies at 103, and antiribonucleoprotein antibodies at 36 (reference value for each measurement, <20). Physical examination at presentation revealed a diffusely erythematous rash accompanied by hyperkeratosis and intense pruritus on the face, scalp, and bilateral hands. The patient's prior immunosuppressive therapies included infusions with rituximab, 1000 mg twice spaced 2 weeks apart, and belimumab at a maintenance dose of approximately 700 mg every 4 weeks, in the preceding 4 months. Laboratory workup showed C3 levels at 81 mg/dL (normal range, 83-177 mg/dL), C4 levels at 19 mg/dL (normal range, 15-45 mg/dL), erythrocyte sedimentation rate at 35 mm/h (normal range, 0-20 mm/h), C-reactive protein levels at 1.4 mg/dL (normal range, 0.0-0.8 mg/dL), and double stranded DNA titer at 1:80 (negative titer); the patient began a prednisone taper and restarted hydroxychloroquine for a presumed lupus flare.

One month later, the patient's rash had not improved, and the dermatology department was consulted. Her pruritus, severe hyperkeratotic scale (Figs 1 and 2), interdigital involvement, and the presence of burrows (Fig 3) raised suspicion for scabies, confirmed by mineral oil preparation examination. The Royal Darwin Hospital clinical grading scale was used to treat crusted scabies. It accounts for the distribution of skin lesions, the extent of crusting, previous scabies diagnoses, and skin condition when determining ivermectin dosage.^2^ The patient began oral ivermectin, 12 mg on days 0, 1, 7, 8, and 14, and 5% permethrin cream therapy, leading to rapid alleviation of her symptoms.

**Fig 1:**
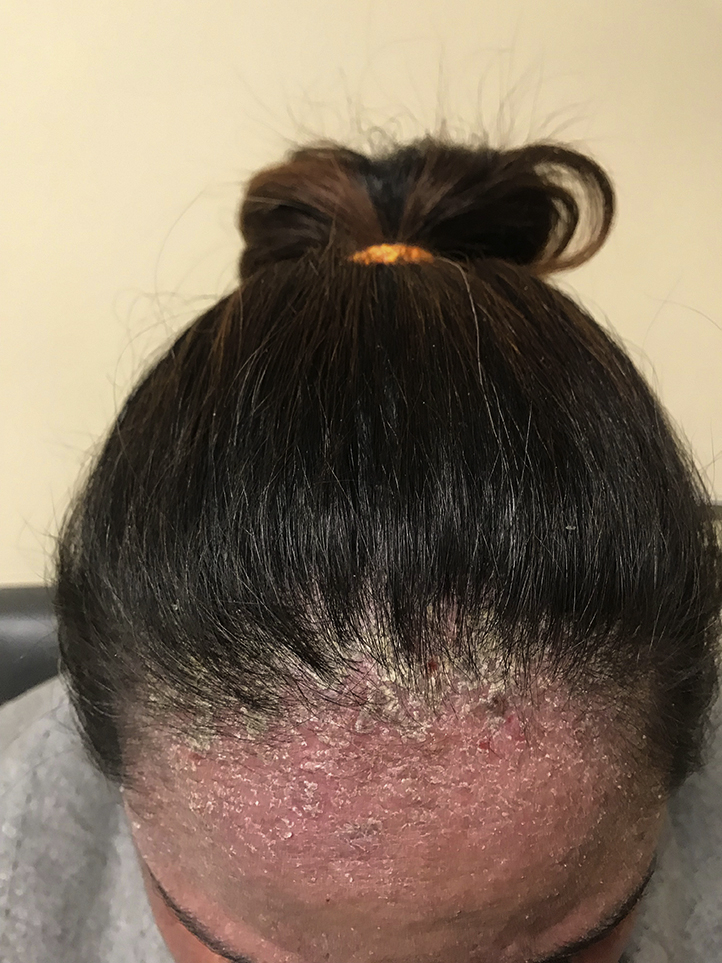
Clinical photograph of the patient's scalp demonstrating scaling of the skin.

**Fig 2:**
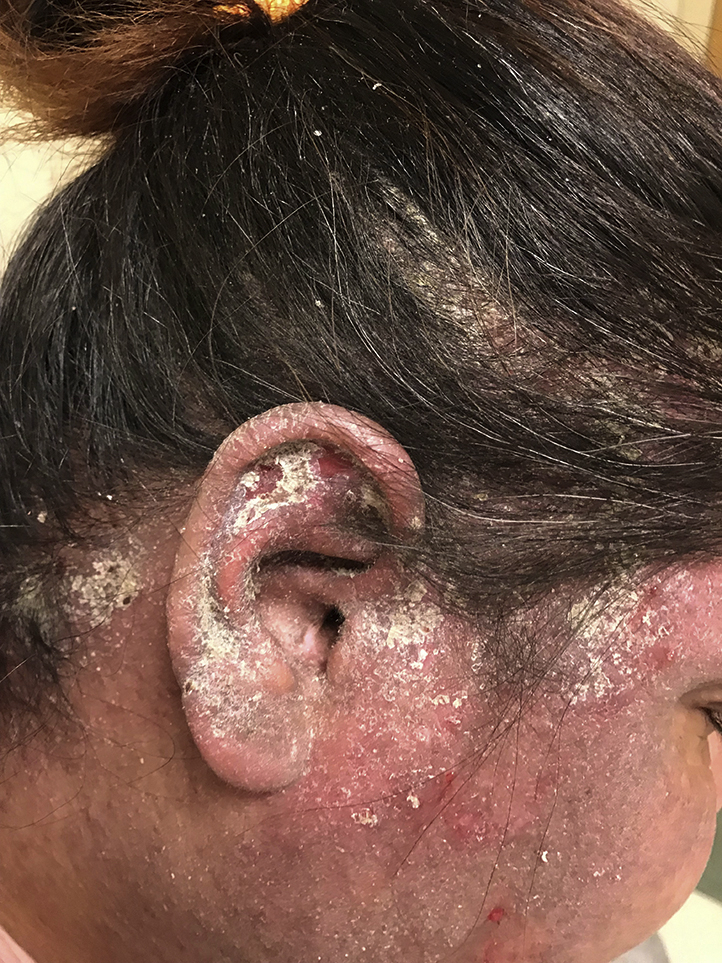
Clinical photograph of the patient's right ear showing crusting due to hyperkeratosis.

**Fig 3:**
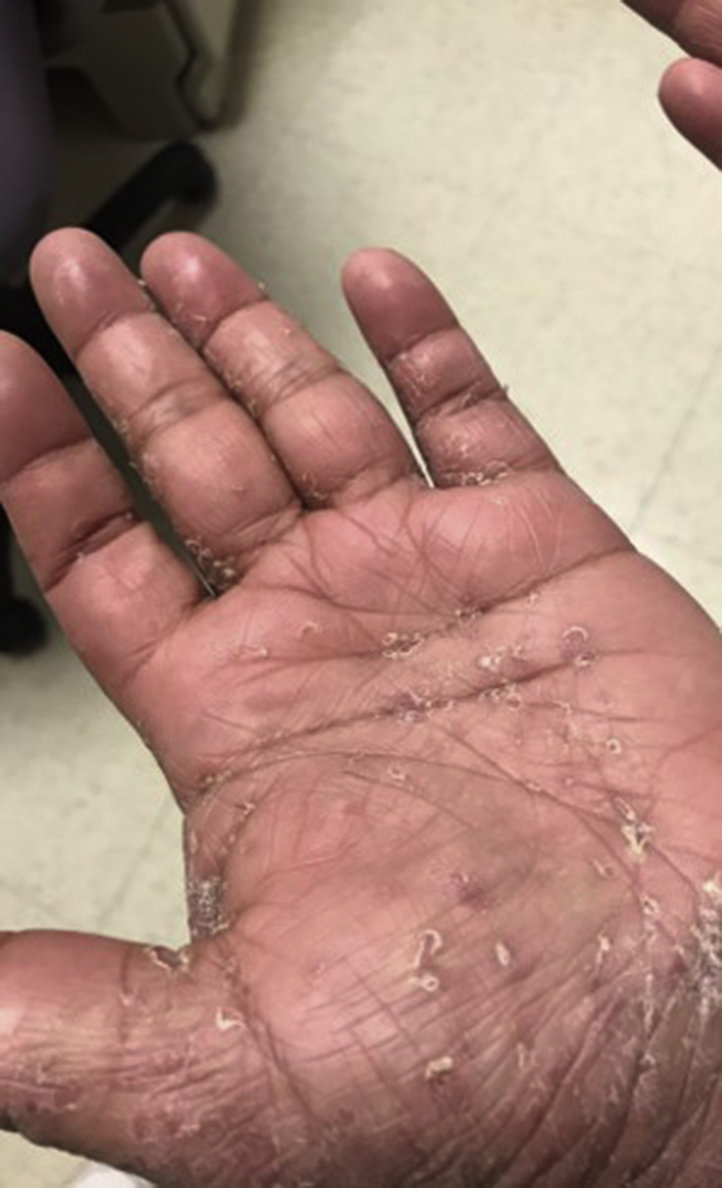
Clinical photograph of the palm, showing crusted plaques and burrows at time of diagnosis.

Our case further suggests a possible connection between increased risk of scabies infection after rituximab infusion, in addition to Ashrafzadeh and Layegh's^1^ observations. This consideration should include patients with SLE who are treated with similar B cell–suppressing regimens, notably belimumab. Belimumab is the only antibody drug that has been approved by the US Food and Drug Administration for the treatment of SLE.^3^ It binds the B lymphocyte stimulator ligand, thereby inhibiting B cell maturation.^3^ Although suppression of B cell function is clinically effective for SLE management, the absence of B lymphocytes in crusted scabies lesions may reflect an ineffective immune response to the infestation.^4^

Scabies can cause diagnostic dilemmas and delay in patients with SLE with immunosuppression. In our case, the erythema and anatomic distribution of lesions on the scalp, ears, and face mimicked the patient's previous lupus flares. However, normal complement levels suggested that lupus was not the cause of her present symptoms. For these reasons, we encourage careful clinical evaluation of any patient who experiences an exacerbation of their cutaneous lupus while on B cell–suppressive therapy. Crusted scabies should be considered before the initiation of immunomodulating agents.

## Conflicts of interest

None disclosed.
